# Environmental assessment of pediatric Lead exposure in Tehran; a prospective cross-sectional study

**DOI:** 10.1186/s12889-021-11494-1

**Published:** 2021-07-21

**Authors:** Hedieh Ahangar, Afsoon Karimdoost, Amir Salimi, Maryam Akhgari, Scott Phillips, Nasim Zamani, Nasibeh Hassanpour, Ali-Asghar Kolahi, Gary R. Krieger, Hossein Hassanian-Moghaddam

**Affiliations:** 1 Pharmaceutical Sciences Branch, Islamic Azad University (IAUPS), Tehran, Iran; 2 School of Medicine, Shahid Beheshti University of Medical Sciences, Tehran, Iran; 3 Legal Medicine Research Center, Iranian Legal Medicine Organization, Tehran, Iran; 4 University of Colorado Anchutz Medical Campus, Rocky Mountain Poison & Drug Safety, Denver, CO and Washington Poison Center, Seattle, WA USA; 5 Social Determinants of Health Research Center, Shahid Beheshti University of Medical Sciences, Tehran, Iran; 6 Department of Clinical Toxicology, Loghman Hakim Hospital, School of Medicine, Shahid Beheshti University of Medical Sciences, South Karegar Street, Tehran, Iran; 7 Skaggs School of Pharmacy and Pharmaceutical Science, University of Colorado Anschutz Medical Campus, Aurora, CO 80045 USA

**Keywords:** Lead toxicity, Children, Trace elements, Plumbism

## Abstract

**Background:**

Ingestion and inhalation are common routes of exposure for lead in humans. Developing countries still have unacceptably high rates of lead toxicity, especially in children. Studies on probable risk factors of lead poisoning in Iranian children are insufficient. In this study, we aimed to evaluate possible environmental factors in children with high blood lead concentrations living in Tehran and neighboring cities.

**Methods:**

In a prospective cross-sectional study between March 2018 and March 2019 we followed all children referred from two pediatric gastrointestinal clinics with blood lead level (BLL) > 5 μg/dL in metropolitan Tehran to investigate possible environmental risk factors in their home. Household specimens including scratched wall paint, house floor dust, windowsill dust, tap water, and consumed spice were evaluated using atomic absorption method to detect lead concentrations. Epidemiological and environmental data collected through in-depth interviews with parents/guardians. Industrial areas were defined based on municipality maps on industrial places.

**Results:**

Thirty of 56 parents/guardians with BLL > 5 μg/dL agreed to be followed through environmental investigation. The only categorical statistically significant risk factor was a history of lead poisoning in the family and living in an industrial zone. There was a positive correlation between BLL and interior windowsills dust lead level, *r* = 0.46, *p* = 0.01. Scratched paint lead level and BLL showed a significant positive correlation, *r* = 0.50, *p* = 0.005. House floor dust lead level (median = 77.4, *p* < 0.001) and interior windowsill dust lead level (median = 291, *p* = 0.011) were both significantly higher than the environmental protection agency (EPA) standards of 40 μg/ft^2^, 250 μg/ft^2^. Interior windowsill dust lead concentrations were significantly higher in industrial areas (*p* = 0.026).

**Conclusion:**

Children’s playing environment should be cleaned more often to reduce dust. Moreover, specific rules may need to be implemented for paint lead concentrations and air pollution, especially in industrial areas.

**Supplementary Information:**

The online version contains supplementary material available at 10.1186/s12889-021-11494-1.

## Background

The physical properties of lead allow this metal to be used in many industrial applications [1]. However, lead has numerous detrimental effects on several organ systems, most notably the nervous system [2]. For years, lead has been utilized in various industries and products leading to higher contact and higher poisoning rates [3]. Lead toxicity is a worldwide public health hazard. There is no completely safe blood lead level (BLL) to date – even 5 μg/dL - and damage may occur at low BLL [4, 5]. Even though developed countries promoted environmental regulations to lower lead poisoning rates, developing countries still represent high rates of lead toxicity, especially in the children [6].

Ingestion and inhalation are the most common routes for lead absorption [7]. In addition to occupational and environmental exposures, drinking water, food [8], spices, traditional medicine, acidic canned foods in lead-soldered containers are the most common foods contaminated with lead [9–11]. Older water pipes and other water infrastructures release lead into the water [12, 13]. Water treatment procedures and changing the water source are other reasons for water lead contamination [14, 15].

Household dust [16]; decayed paints, soil dust near roads or industrial areas, lead-glazed ceramics, home remedies, cookware and air pollutants are probable house dust sources [17]. Urbanized residential topsoil show about 7 times higher lead concentration. There are difference between sampling locations and the highest attributed to dripline at the same area [18]. Lead remains in topsoil for even centuries and its redistribution exposes people to higher toxicity risk [19].

Lead affects almost all organ systems in the body, including the nervous, digestive, renal, and hematopoietic systems [20, 21]. Lead toxicity is mostly without any signs or symptoms. The most common signs or symptoms are constipation, abdominal pain, anemia, fatigue, weakness, irritability, renal dysfunction, and neuropathies [2, 21]. Neurological symptoms like learning impairment, irritability, attention-deficit/ hyperactive disorder, and lower IQ scores are identified in children who may also have poor socio-economic support, reinforcing the importance of lead poisoning [22–25].

Children may absorb proportionally more lead than adults and excrete less lead in urine [26, 27]. Children crawl on the floor, have hand-to-mouth and pica behaviors, all of which make them at higher risk of exposure to lead [28]. After knowing more about lead’s adverse effect on children’s developing brain, a couple of regulations are implemented to decrease lead exposure. Governments phased out leaded gasoline, defined limits for maximum lead in paints, and drinking water [29–31]. In Iran, leaded gasoline was eliminated in 2000. With lead removal from petrol, there has been a sustained decline followed by a plateau.

Studies on probable risk factors of lead poisoning in Iranian children are insufficient. In this study, we aimed to evaluate possible environmental factors in children with high BLLs living in Tehran and neighboring cities.

## Methods

### Study design & settings

This cross-sectional study includes children referred from two pediatric gastrointestinal clinics with BLL > 5 μg/dL in Tehran between March 2018 and March 2019.

### Sample size and participants

A previous study at one of two workplaces showed a BLL > 5 μg/dL in 25% of referral patients to pediatric clinic [32]. The following formula is used to calculate the size of the required sample:

$$ \mathrm{n}=\left(\mathrm{z}\right)2\ \mathrm{p}\ \left(\ 1-\mathrm{p}\ \right)/\mathrm{d}2 $$

**Fig. 1:**
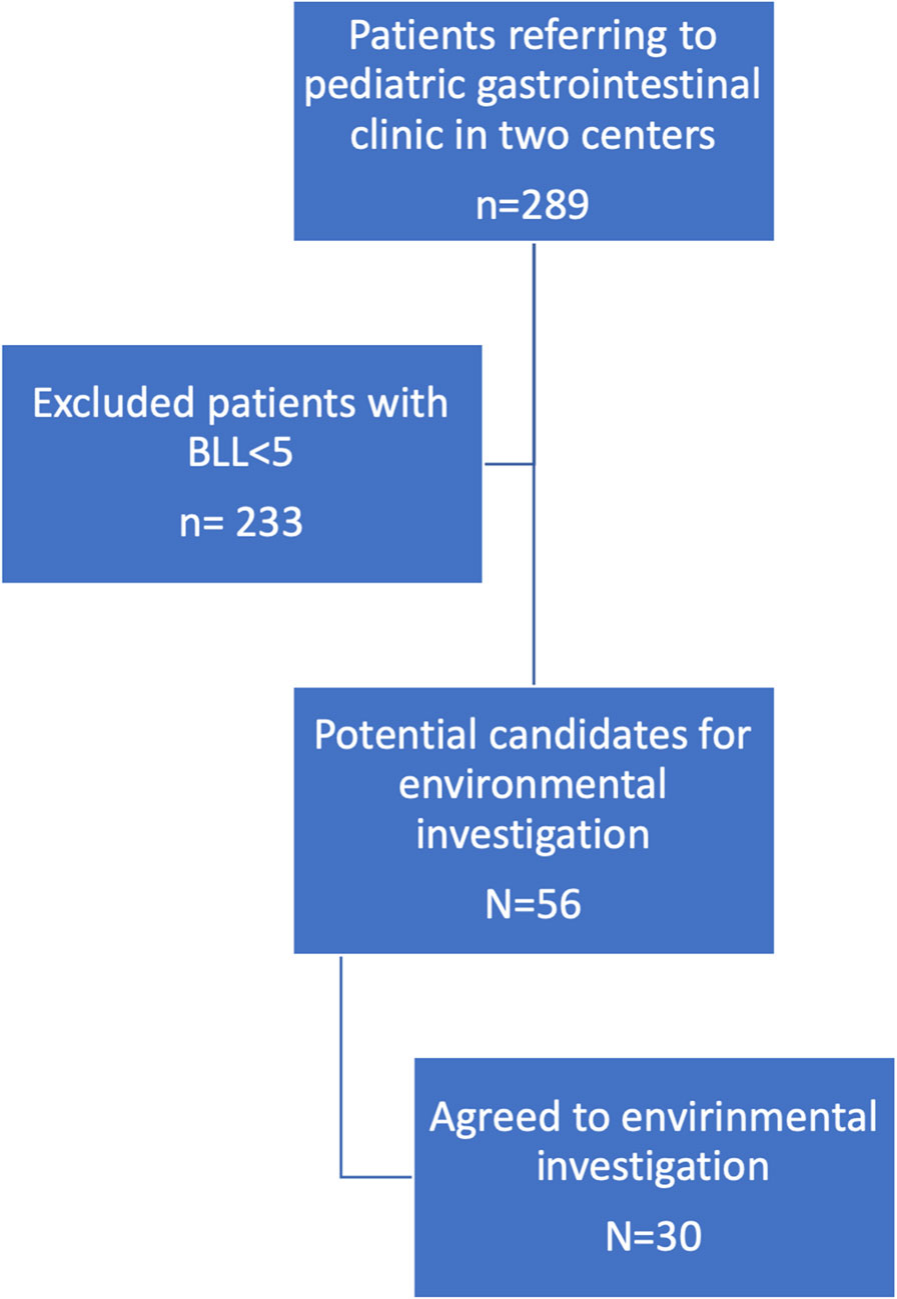
Flowchart of selecting patients for environmental assessments

Considering 95% confidence interval (z = 1.96) and tolerated margin of error equal to 0.5, the required sample size was 289 cases. These participants were chosen based on a convenience sampling from all pediatric population that referred due to abdominal pain and/or constipation to gastroenterology clinics. Inclusion criteria were defined as 1- BLLs higher than 5 μg/dL. 2- Agreeing to participate in an interview and allowing essential lab studies. 3- Agreeing to allow environmental investigations including sampling in the home. The only exclusion criterion was parents (guardians) unwillingness to participate in the study. Figure 1 shows the flowchart of recruitment.

Patients were tested for BLL by voltammetry method. Patients with BLLs higher than 5 μg/dL were included in the final environmental evaluation of measuring household specimens lead concentrations with atomic absorption method.

### Variables

Parents reported demographic data (Supplementary questionnaire). Stature-for age were calculated and categorized based on WHO child growth standards. BMI-for-age were calculated and categorized based on WHO growth reference charts and tables. For children under 5 years of age: overweight is defined as weight-for-height greater than 2 standard deviations (SD) above WHO Child Growth Standards median; and obesity is defined as weight-for-height greater than 3 SD above the WHO Child Growth Standards median. For between 5 and 19 years children, overweight is defined as BMI-for-age greater than 1 SD above the WHO Growth Reference median; and obesity is defined as greater than 2 SD above the WHO Growth Reference median [33]. Parents were asked about children age, restlessness history, living in an area known as industrial, history of lead poisoning in family, pica, house age, residence in house duration, water pipe material and age, tap material and age, recent house painting, and consuming excessive spice compared to relatives and friends. Environmental samples were gathered to measure their lead concentrations by atomic absorption method.

### Sampling & measurement

To evaluate environmental factors, scratched wall paint, house floor dust, windowsills’ dust, tap water, and consumable spice with lead contamination were evaluated.

Wall paint specimen, scratched by a stainless steel scalpel in four different places where the child spends more time, was obtained [34]. Scratched paints were stored in a plastic bag in 4 °C until lead level measurement. One gram of paint was dissolved in 5 mL nitric acid and 15 mL hydrochloric acid (aqua regia) to measure the paint lead level. Then the solution was kept 30 min in room temperature under hood. Then it was kept in a heater with a temperature of 50 °C for 30 min. After that, the solution was cooled to reach room temperature. The solution was filtered using Whatman filtration paper. Finally, lead concentrations were measured by graphite furnace atomic absorption spectrometry (GFAAS) method with Agilent Technologies GTA120 Graphite Tube Atomizer device [34].

Two places in which the child spends the most time were evaluated for the dust lead level. Floor dust was sampled by wiping a one-foot square area of the house floor with a wet wipe. We wiped the surface with an S-shaped pattern to ensure a complete cleaning. The process was repeated for one more time, and the specimen was stored at 4 °C until evaluation for lead level. Then one gram of the wet wipe dissolved in aqua regia; the remaining procedure is the same as for scratched paint [35].

Child’s room windowsills dust obtained from an area of 100 cm^2^ (2 by 8 in.). Dust wiped by a wet wipe in a linear direction three times on the targeted area. All other procedure parts are the same as floor dust evaluation [35].

The most frequently used self-reported spice in each family in cooking were sampled in each house to measure lead concentrations. To measure spice lead concentrations, one gram of spice was dissolved in aqua regia, as previous specimens. The same method was used to measure spice lead concentrations. Tap water was sampled for measuring household consuming water lead level. The water lead level is directly measured using atomic absorption method.

### Statistical analysis

We stratified the demographic characteristics and investigated differences in their BLLs. Due to the violation of normality assumptions (based on Shapiro wilk test and generated Q-Q plot) in BLLs, we performed Mann-Whitney and Kruskal Walis test to determine the relation between BLLs and demographic characteristics as probable risk factors. Pearson’s correlation was performed to evaluate the relation between continuous data and BLL. All univariate analyses which showed a *P* < 0.05 were included in the final fitted multivariate regression model to quantify the relationship between them and BLL. A *p* < 0.05 considered significant in the final analysis. One sample Wilcoxon signed-rank tests were conducted to compare paint lead, water lead level, surface dust lead level, and interior windowsill with international standards.

## Results

**Fig. 2:**
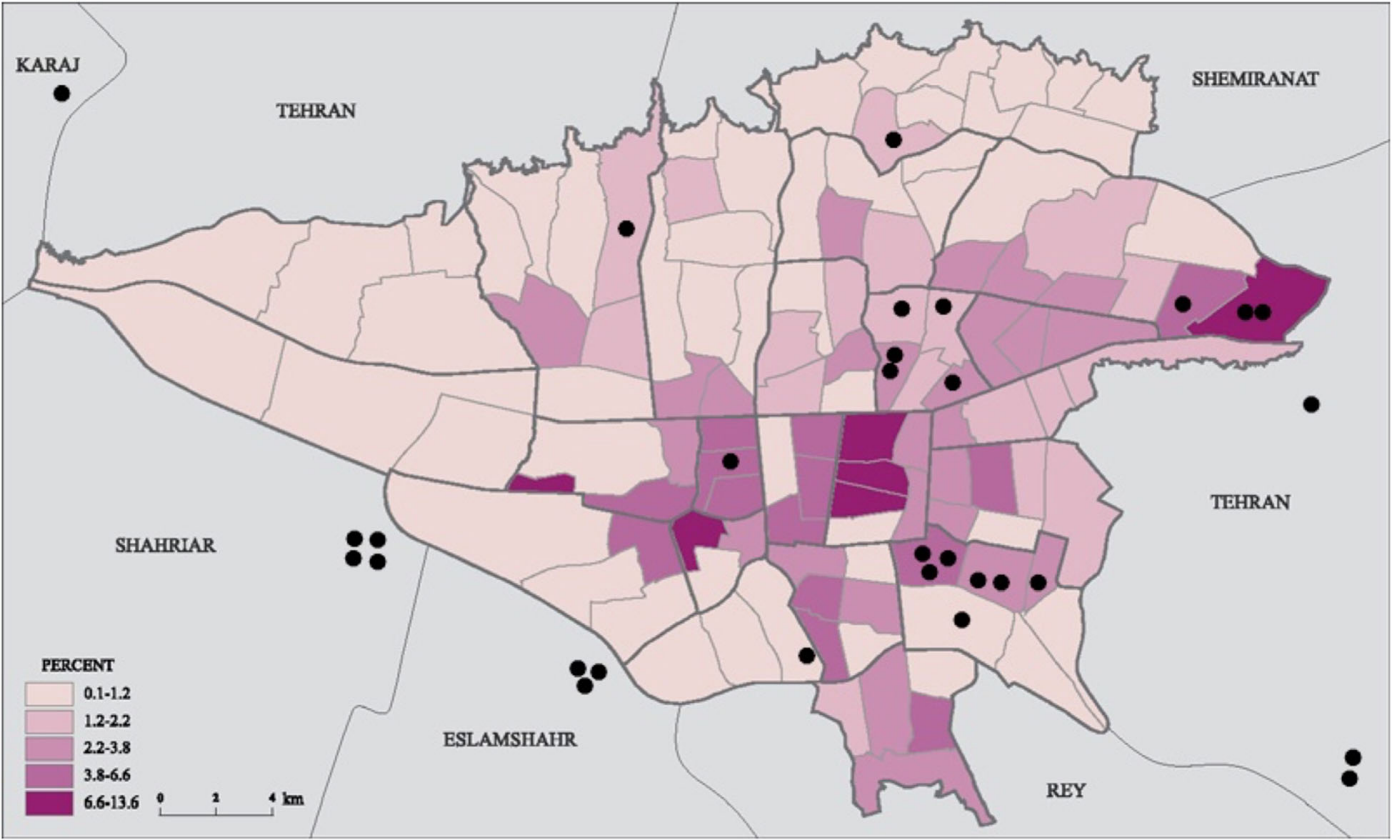
Tehran Geo-mapping industrial districts and referral pediatric patients (Industrial zones are retrieved from Tehran Municipality map: https://atlas.tehran.ir/Default.aspx?tabid=318)

During the study period, 56 cases with BLL > 5 μg/dL from two different pediatric gastrointestinal clinics referred for environmental follow-up. Of whom 30 (53.6%) agreed to be followed through environmental investigation. Figure 2 depicts the geolocations of study subjects living locations in Tehran, with considering adjacent industrial districts.

The final participants’ mean age was 6.1 years (SD = 3.1, range 1–13 years). Participants’ BLL based on their characteristics are presented in Table 1 and Table 2. The only categorical statistically significant risk factor was a history of lead poisoning in the family and living in an industrial zone.

**Table 1:** Demographic and clinical characteristics

Demographic and clinical characteristics		N	Median BLL (IQR)	*P*-value
Sex	F	19	8.9 (6.2–24)	0.698
	M	11	6.9 (5.5–25	
Age	< 4 y	11	6.9 (5.9–30.6)	0.842
	4–7 y	6	11 (6.4–19.1)	
	> 7 y	13	8.9 (5.3–23.6)	
Stature-age	Severely stunted	2	7.2 (NA)	0.295
	stunted	1	30.6 (NA)	
	Normal	27	8.9 (5.5–24)	
BMI-age	Severely underweight	1	5.3 (NA)	0.71
	Underweight	6	5.8 (5.1–6.8)	
	Normal	17	12.9 (6.5–26.3)	
	Moderately overweight	3	23.2 (NA)	
	Overweight	3	9.2 (NA)	
Pica	no	23	8.5 (5.9–25)	0.787
	yes	5	23.2 (5.3–27.5)	
Food allergy	no	25	8.5 (5.5–20.2)	0.277
	yes	5	23.2 (6.2–45.3)	
Restlessness	no	28	8.7 (5.9–23.8)	1
	yes	2	25 (NA)	
Family history of lead poisoning	no	25	6.9 (5.5–14)	0.008
	yes	5	27.1 (20.2–29.7)	
Addiction in father	no	25	6.9 (5.7–24.5)	0.867
	yes	5	8.9 (6.8–21.7)

**Table 2:** Environmental characteristics

Environmental characteristics		N	Median BLL (IQR)	*P*-value
Reconstruction in 6 month	no	29	8.5 (5.7–23.6)	0.326
	yes	1	25 (NA)	
Building painting in 6 month	no	26	8.7 (5.8–23.6)	0.807
	yes	4	15.1 (5.5–28.9)	
Industrial zone	no	15	6.2 (5.3–12.9)	0.038
	yes	15	16.4 (6.8–27.1)	
Building’s paint type	Oil	26	8.8 (5.8–24.2)	0.737
	Plastic (acrylic)	4	7.5 (5.3–25.1)	
Building’s Pipe material	Metal	14	12.1 (6–25.9)	0.352
	Non-metal	15	6.8 (5.5–12.9)	
	both	1	25 (NA)	
Habit of excessive Spice consumption in family	No	28	9 (5.9–24.7)	0.157
	Yes	2	5.7 (NA)	
	both	1	25 (NA)	
Spice Packaging	Hygiene Pack	6	24.1 (6.7–26.9)	0.280
	Bulk-sale	16	7.9 (5.6–14.5)	
	Both	8	7.6 (5.2–28.9)	
Residence duration	0–3 y	18	8.7 (6.1–24.4)	0.990
	4–6 y	5	6.2 (5.5–44.1)	
	> 7 y	7	11.2 (5.2–25)	

Median (IQR) of BLL, tap water lead, interior windowsills dust lead, house ground dust lead, scratched paint lead, spice lead concentrations are presented in Table 3.

**Table 3:** Specimens lead level

Lead level	Blood (μg/dL)	Tap water (μg/L)	Interior windowsill dust (μg/ft2)	House ground dust (μg/ft2)	Scratched paint (ppm)	Spice (μg/Kg)
Median (IQR^a^)	8.7 (5.8–24.2)	63.5 (36–78.9)	291 (231.2–345.2)	77.4 (66.4–84)	105.6 (71.1–123.7)	and 56.7 (20.6–98.5)

^a^*IQR* interquartile range

**Fig. 3:**
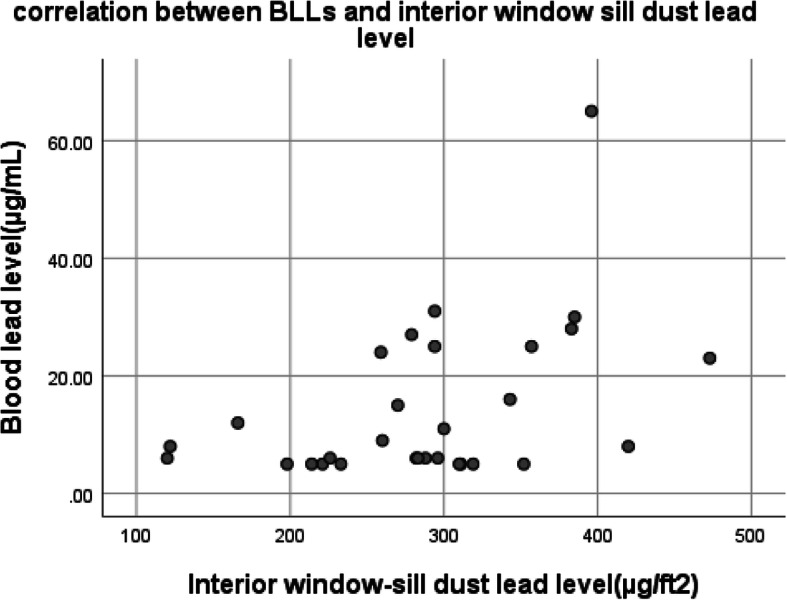
Correlation between BLL and interior window-sill dust lead level

**Fig. 4:**
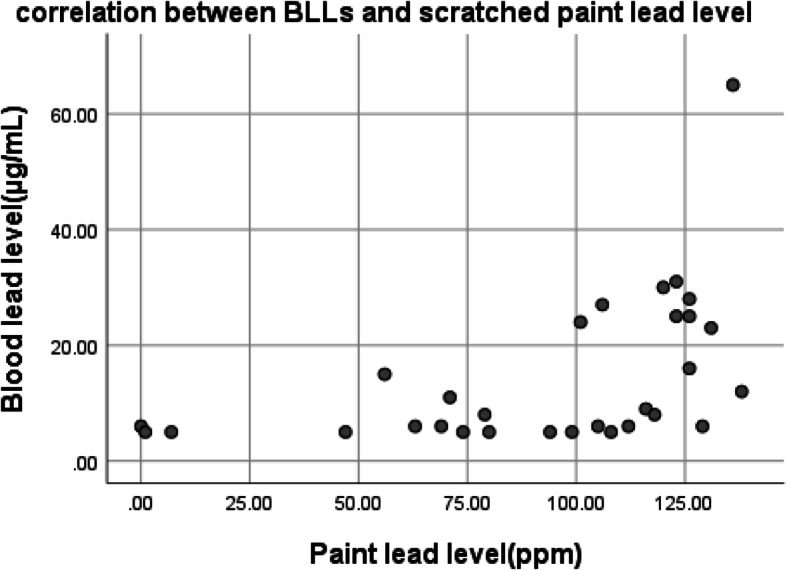
Correlation between BLL and scratched paint lead level

There was a positive correlation between BLL and interior windowsills dust lead level, *r* = 0.46, *p* = 0.01 (Fig. 3). Scratched paint lead level and BLL showed a significant positive correlation, *r* = 0.50, *p* = 0.005 (Fig. 4). No correlation was observed between BLL and participants age (*p* = 0.9), weight (*p* = 0.7) building age (*p* = 0.2), pipe age (*p* = 0.2), water lead (*p* = 0.7), house floor lead (*p* = 0.8), BMI, (*p* = 0.7), and spice lead (*p* = 0.5).

An adjusted multivariate linear regression was calculated to predict participants’ BLL. A variable that showed a significant difference in BLLs in univariate analysis was included in the model; interior window-sills dust lead level, scratched paint lead level, history of lead poisoning in family members, and residence in industrial area were included in the model. A significant regression equation was found (F(5,24) = 4.154, *p* < 0.01), with an R^2^ of 0.464.

The paint lead’s median was not significantly different from the recommended limit of 90 ppm by the U.S. Consumer Product Safety Commission (CPSC) (median = 105.6, *p* = 0.28). However, house floor dust lead level (median = 77.4, *p* < 0.001) and interior window-sill dust lead level (median = 291, *p* < 0.001) were both significantly higher than the environmental protection agency (EPA) standards of 40 μg/ft^2^, 250 μg/ft^2^. Interior window-sill dust lead concentrations were significantly higher in industrial areas (*p* = 0.026).

We compared environmental lead concentrations with corresponding international standards (Table 4).

**Table 4:** Lead concentrations compared with international standards

Lead level	Median (IQR)	Standard limit	*p*-value
Scratched paint (ppm)	105.6 (71.1–123.7)	90 (CPSC^a^)	0.28
House floor dust (μg/ft^2^)	77.4 (66.5–84)	10 (EPA^b^)	< 0.001
Interior window-sills dust (μg/ft^2^)	291 (231.2–345.2)	100 (EPA^b^)	< 0.001
Tap water (μg/kg)	63.5 (36–78.9)	15 (EPA^b^)	< 0.001
Spice (μg/kg)	56.7 (20.6–98.5)	300 (WHO^c^)	< 0.001

^a^ Consumer product safety commission

^b^ Environmental protection agency

^c^ World health organization

## Discussion

This study aimed to identify the responsible sources of lead exposure in children in Tehran, Iran, referred from pediatric gastrointestinal clinics. CDC mentions that lead-based paint and lead-containing dust are the most common and dangerous lead poisoning sources in young children [36]. Our key findings showed that scratched paint lead concentrations and interior windowsills lead concentrations correlate with children’s elevated BLL. Lead concentrations in windowsills dust were significantly higher in participants living in industrial areas.

Until recently, windows in Iranian buildings were not appropriately sealed due to low energy prices [37]. Unsealed windows may pass higher amounts of dust originating directly from polluted air or redistribution of topsoil containing lead [38]. Topsoil lead is a major contributor to childhood lead exposure especially in droughty times [39]. Interior windowsill dust may represent outside rather than inside sources such as leaded paint used on sliding window frames. Our results demonstrated that windowsill dust was the most correlated factor with elevated BLL. Those are in line with Zamani et al. findings that revealed residence in an industrial area with a polluted atmosphere might increase lead poisoning risk. However, in their study, contrary to our results, pipe type and materials affected children’s BLLs [32].

Leaded gasoline as a renowned environmental pollutant phased-out years ago in Iran [32], and atmosphere lead pollution probably originate from other sources like particles emitted from tires, brakes, metal equipment in industrial areas [40]. The roads may matter as this is airborne from auto exhaust but also fine particles on dust again from exhaust that re-entrains on dust and is inhaled or even ingested plus the lead tends to accumulate on floors, yards etc. where children may play, especially children still crawling and have hand to mouth behavior, in ages < 18–24 months. In Iran, children usually start schooling at age six. We could not find any statistical difference between children at schooling years vs. below schooling year. Facing more elevated BLL at higher ages this suggests a different source than soil/dust (i.e., maybe water (pipes), possibly food but more commonly it is an active air source from somewhere- mainly roads and industrial processes.

A few years ago, cars used leaded gasoline and released large amounts of lead into the air. Released lead finally settled on the city ground; redistribution of settled lead into the air might be another source of air lead pollution entering houses through windows [37, 41]. Moreover, Taghavi et al. analyzed streets’ dust lead concentrations a few years after phasing out leaded gasoline. They showed that Tehran streets are moderately polluted by lead based on the geo-accumulation index (3 < Enrichment Factor (EF) ≤ 5; Mean pb = 110.27) [40].

House paint lead concentrations were marginally correlated with participants’ BLL. However, paint lead concentrations in our study were not significantly higher than the CPSC standard of 90 ppm [40]. We hypothesized that it might be due to the lower quality of paints in Iran, which results in higher rates of peeling and cracking. All of which results in more lead paint chips and dust, making children susceptible to lead poisoning even if the paint’s lead did not exceed international standards. Paint lead concentrations usually were correlated to children’s BLLs in conducted studies on this matter that is in line with our results [16, 26]. Recent painting and reconstruction showed no significant effect on children’s BLLs.

Even though the measured house floor’s dust lead concentrations were significantly higher than EPA standards [42], they were not significantly correlated with elevated BLLs. Children crawling on the floor are the most in-contact to floor dust lead ingestion and poisoning [43]. Participants in our study were older than 1 year and probably able to walk and crawl less on the floor. So, despite high lead concentrations of floor dust, it was not correlated to BLLs.

Spice lead concentrations were not higher than WHO standards [44] and were not correlated to BLLs. Both of which reinforces the unlikelihood of spice-induced lead poisoning. Our results on the Tehran water lead level, were in accordance with previous Iranian studies, and suggest that Tehran tap water contains more lead than EPA standard [45–48]. However, it was not correlated with higher BLLs. Higher BLLs were not related to having more food sensitization which is in accordance with Mener’s results that showed no significant change in food allergic sensitization rates in children with high BLLs unlike adults [49].

Lead poisoning has irreversible effects on brain development and children’s height leading to high burdens on health systems [15, 25, 50, 51]. We did not notice a significant decrease in participant’s height and mental function. However, 2 had hyperactivity disorder.

Geo-mapping is helpful to find lead hot spots for cleanup or further cases of lead poisoning. We were not able to do soil mapping but could define industrial districts on a simple map to look at patterns of exposure.

## Conclusion

To avoid the burden of lead poisoning, parents should be informed about the most common reasons and sources of poisoning; children’s laying environment should be cleaned more often to reduce dust. Moreover, specific rules should be implemented about paint lead concentrations and air pollution, especially in industrial areas.

### Limitations

Our study’s most significant limitation was sample size; lead poisoning is not highly prevalent in Tehran, making it more difficult to recruit more participants who accept to be assessed in the environment. We suggest that researchers conduct multicenter studies with bigger sample sizes. A major limitation is the lack of soil Pb results in the neighborhoods of the industrial zone compare to the other parts of the city. This could yield important information about the source of Pb dust in Tehran. Another limitation is the lack of a control group in this study. So, we were not able to investigate the kids’ environment with normal BLL.

We suggest performing another study including a control group to develop more robust results.

## Supplementary Information

**Additional file 1.** Environmental lead assessment questionnaire.

## Data Availability

The datasets used during the current study available from the corresponding author on reasonable request.

## Abbreviations

BLL: Blood lead level
CDC: Center for disease control
CPSC: Consumer Product Safety Commission
EPA: Environmental Protection Agency
GFAAS: Graphite furnace atomic absorption spectrometry
WHO: World health organization
