# Supplementary material for: Environmental assessment of pediatric Lead exposure in Tehran; a prospective cross-sectional study
Source: BMC Public Health. 2021 Jul 21;21:1437. doi: 10.1186/s12889-021-11494-1 (PMC8296531; doi:10.1186/s12889-021-11494-1)
Supplement: Supplementary file 1 — Additional file 1. Environmental lead assessment questionnaire. [file 12889_2021_11494_MOESM1_ESM.pdf]

Environmental lead assessment questionnaire

Dear parents/guardians, please fill the questionnaire about your child and house carefully based on the instruction gave to you. Filling this questionnaire and/or participating this study is not compulsory and you should be informed about the whole process before consenting to enroll.

Child name:	
Age:	
Gender:	
Weight:	height:
Residence duration at the building:	
Recent building reconstruction (last six months): yes no	
Recent building painting (last six months) : yes no	
Building age:	
Building painting type:	
water pipe material:	
water pipe age:	
Building tap material:	
Building tap age:	
Living district name:	Living district municipality name:
Are you living in an industrial area?	If yes, what is the industry?
What is the material for child's usual toys?	
Father's job:	
Mother's job:	
Does the child have babysitter at house?	If yes what is her another job?

Does anyone living at your house is addicted to drugs? If yes please name his/her kinship, drug type, addiction duration, and consumption route.
Pica habit (eating unusuals and weirds like ice, dust, chalk and etc):
History of food allergy: yes no
Attention deficit: yes no hyperactivity disorder: yes no restlessness: yes no
Spice type used: bulk-sale hygiene packaging
The most frequently used spice:
Spice consumption in each month:
History of lead poisoning in family: yes no
Eating habit disorders: yes no
Abdominal pain: if yes, duration:
Constipation: if yes, duration:

Name: date and sign:
